# Validation of the brief instrument “Health Literacy for School-Aged Children” (HLSAC) among Norwegian adolescents

**DOI:** 10.1038/s41598-022-26388-4

**Published:** 2022-12-21

**Authors:** Hanne Nissen Bjørnsen, Unni Karin Moksnes, Mary-Elizabeth B. Eilertsen, Geir Arild Espnes, Gørill Haugan

**Affiliations:** 1 Department of Public Health and Nursing, Norwegian University of Science and Technology, Postbox 8905, 7491 Trondheim, Norway; 2 NTNU Center for Health Promotion Research, Norwegian University of Science and Technology, 7491 Trondheim, Norway; 3 Faculty of Nursing and Health Science, Nord University, 7601 Levanger, Norway

**Keywords:** Health care, Disease prevention

## Abstract

Health literacy (HL) has been identified as an important and modifiable determinant of health. To succeed in promoting HL, it is crucial to evaluate interventions addressing HL using validated instruments. However, HL measurement is an under-researched area among adolescents. The Health Literacy for School-Aged Children (HLSAC) scale is developed in Finland to measure the subjective HL levels of school-aged children. The HLSAC has been used and validated across Europe. No validated instrument for measuring HL among Norwegian adolescents has been identified. Therefore, the aim of this study was to assess the psychometric properties of the HLSAC among Norwegian adolescents in upper secondary schools. Cross-sectional data were collected in 2017 from 1 054 adolescents with a response rate of 93.5%. The participants were students aged 16–21 (mean = 17.3) years from four upper secondary schools in an urban area in Mid-Norway. Confirmatory factor analysis (CFA) was utilized to investigate the underlying dimensionality of the data together with composite reliability based on Raykov’s reliability coefficient and certain aspects of construct validity. The original ten-item one-dimensional version of the HLSAC revealed a poor fit. A one-dimensional version including six of the original ten items presented the best fit to our data, demonstrating good reliability and construct validity. This validation study suggests a one-dimensional solution of the HLSAC scale including six of the original ten items, i.e., the HLSAC-6 as a statistical valid and reliable measure for HL among adolescents in upper secondary schools. However, the modifications of the instrument indicate the need for further investigation of the HLSAC in this age group, i.e. by conducting cognitive interviews and focus-group discussions on the concept of HL among adolescents. Testing the wording of the HLSAC prior to additional psychometric evaluations of both the original HLSAC and the suggested HLSAC-6 is recommended. Finally, developing more age-appropriate items for the measurement of HL in this age-group is suggested.

## Introduction

Over the last decades, there has been a growing interest in the concept of health literacy (HL) among children and adolescents^1–3^. This interest may be motivated by an escalating awareness of HL as an important and modifiable determinant of health and, moreover, HL’s potential to benefit child and adolescent present and future health^4–6^. HL has been linked to greater empowerment, health equity and the achievement of the United Nations Sustainable Development Goals (SDGs) thus, signifying HL as an enabler for developing better health and well-being over the life course^4,5^.

Personal HL, as defined by the Centers for Disease Control^7^, refers to “*the degree to which individuals have the ability to find, understand, and use information and services to inform health-related decisions and actions for themselves and others*”. The work of defining and operationalizing HL is imperative for valid and reliable measurement tools, where several definitions and models of HL have been introduced, depicting HL as a multidimensional and complex construct^3,8^. The UN Convention on the Rights of the Child declares that children have the right to receive health information: the UN convention points out that all children must have information on how they can stay safe and healthy. Furthermore, children are required to receive that information in an age-appropriate manner^9^. Children and adolescents thus have specific rights recognizing their HL needs.

Adolescence is described as an important and critical transitional period in life associated with challenges as well as opportunities for growth and development; hence, it is an influential period in life important for addressing HL^10^. Therefore, HL is considered a key outcome of health education interventions, for which children and adolescents are seen as particularly important target populations^4,11,12^. Moreover, solid evidence suggests that a foundation for various health behaviors is established during childhood and adolescence, leaving great potential for health promotion and public health interventions addressing HL during this period in life^4,10,11^. In 2019, the Norwegian government published a strategy to increase HL in the Norwegian population^13^. Additionally, Norway's action plan to achieve the UN's SDGs by 2030 describes under sustainability goal #3 that to ensure good health and wellbeing for all regardless of age, the strategy to increase HL in the population should be addressed nationally. To address and improve HL, it is crucial to assess HL as well as evaluate interventions and working strategies targeting HL using validated instruments. Despite the increased attention in HL over the last decades, HL measurement among children and adolescents remains an under-researched area, and few brief and generic instruments are available^14^. Compared to the number of validated HL instruments for adults, significantly fewer measurement tools are available for younger age groups^15^.

The Health Literacy for School-Aged Children (HLSAC) scale is a self-administered instrument developed in Finland to measure the subjective HL level of school-aged children^11^. The HLSAC includes two items from each of the following five components: theoretical knowledge, practical knowledge, critical thinking, self-awareness, and citizenship^11^. Five of the ten items were informed by the widely-used Health Literacy Questionnaire (HLQ)^16^. Both a 5-factor model and a one-dimensional model were originally tested, with a one-dimensional solution for the HLSAC preferred in Finland^11^.

The HLSAC has been used across Europe and validated in Slovakia and Belgium^17^, Italy^18^, Poland^19^, France^20^, Denmark^21^ and Turkey^22^. No validated instrument for measuring HL among Norwegian children and adolescents has been identified. A recent validation study comparing three measurement scales for HL among adolescents, namely, the HLSAC, the Health Literacy Assessment Scale for Adolescents (HAS-A) and the 16-item European Health Literacy Survey questionnaire (HLS-EU-Q16), supported the use of the HLSAC to assess HL during adolescence^23^. The HLSAC has been used in previous studies in Norway^24,25^; however, no studies have investigated the psychometric properties of the Norwegian version of the measure. In accordance with the Standards for Education and Psychological Testing^26,27^, this study aims to request evidence related to the dimensionality, reliability, and construct validity of the HLSAC, which are considered interrelated measurement properties.

## Methods

### Aims

The aim of this study was to assess the psychometric properties of the Norwegian version of the HLSAC scale among adolescents aged 16–21 years. A scale’s psychometric properties relate to its dimensionality, reliability, and construct validity, all of which are considered interrelated measurement properties. *Dimensionality* is concerned about the homogeneity of the items^28^ examining if the items match the defined construct, which in the present study is “Health Literacy for School-Aged Children”. *Reliability* encompasses an instrument’s internal consistence and lack of error variance^26,28^. We used the reliability coefficients Cronbach’s alpha (α) and Raykov’s reliability (ρc) to assess internal consistence of the items. In this study, c*onstruct validity* denotes if the HLSAC measures the construct it is proposed to measure. Construct validity is based among others on the constructs’ relationships to other variables and constructs^28^. Convergent validity is a supporting piece of evidence for construct validity, testing that related constructs correlate in the expected direction. *Content validity* is embedded in evaluation of construct validity and refers to the degree to which an assessment instrument is relevant to, and representative of, the targeted construct it is designed to measure^29^. If the wording of items is too similar, the reliability coefficients (alpha and composite), content validity and dimensionality will be falsely improved; that is, the average correlation among items increases and therefore also the reliability coefficients, however without adding substantially to the content validity of the scale. Certainly, to tap into the construct some similarity among the items is needed. Even so, items representing merely a rewording of other items are undesirable since they contain limited new information about the construct^30^. Accordingly, theory, validity, reliability, and dimensionality are entwined.

Based in previous research the dimensionality of the HLSAC seems to be uncertain; the scale has five domains indicating a five-factor structure which was tested in Finland^11^; still, a unidimensional solution has showed the best fit^11^. Therefore, this study aimed to answer the following three questions: (a) How well does the original one-factor measurement model of the HLSAC fit to the observed data? (b) Does the 5-factor model tested in Finland and Italy fit better? (c) Does the HLSAC reveal good reliability and construct validity among Norwegian adolescents 16–21 years old? Concerning convergent validity (an aspect of construct validity), we expected the HLSAC to correlate with some established concepts (hypothesis 3). The following three hypotheses (H_1_, H_2_, H_3_) were stated:

#### Hypothesis 1 (H_1_)

The one-factor model of the Norwegian HLSAC provides a better model fit than the five-factor model.

#### Hypothesis 2 (H_2_)

The Norwegian version of the HLSAC shows good reliability and construct validity among Norwegian adolescents 16-21 years old.

#### Hypothesis 3 (H_3_)

: The HLSAC correlates positively with positive mental HL, and adolescents’ perceived level of knowledge needed to take care of their own health.

### Participants and procedure

The study participants included a cross-sectional sample of adolescents aged 16–21 years in an urban area in Mid-Norway with a mean age of 17.3 years old. Data were collected via a survey questionnaire in four upper secondary schools in 2017. The schools represent typical Norwegian upper secondary schools in an urban area of Norway. The school sizes varied from 260 to 1 087 students. The schools’ principals gave informed consent for data collection at the designated schools. The questionnaires were available toward the end of the 2017 school year, from April-June, during which time the teachers chose a convenient 45-min session for survey administration. The questionnaire was distributed class-wise at the time of the class teachers` choosing, during a three-month period where the survey was available to pick up at the teachers’ lounges at the participating schools. The questionnaires were administered using pen and paper, and students could choose to do schoolwork if they decided to not participate in the study.

### Ethical approval and consent for participation

Prior to the survey, principals, and teachers at each of the four included schools received information about the study from the research group, in writing and over the phone. Information about the study was provided to students and parents through a written hard copy invitation letter and through an informational video available at the schools’ e-learning platforms. Participants were aged 16 years and older and gave an informed consent (according to Norwegian Law) for participation by completing the questionnaire. Participation was voluntary and anonymous. The study was approved by the Regional Committee for Medical and Health Research Ethics (REK midt 2014/1996). All steps of the study has been performed in accordance with the Declaration of Helsinki.

### Measures

#### HLSAC

The HLSAC is a one-dimensional instrument developed in Finland in 2016. HLSAC consists of 10 items measuring subjective HL among school-aged children assessed on a four-point Likert scale, ranging from *not at all true*, *not quite true, somewhat true* to *absolutely true*^11^. Originally, ten items were developed and tested among Finnish 13- and 15-year-olds. The scale has been reported to be suitable for use in monitoring children’s and young people’s HL^11,12,18–23^. The ten items cover five core components of HL: theoretical knowledge, practical knowledge, critical thinking, self-awareness, and citizenship^11^. Based on these five theoretical components, Pakkari et al.^11^ tested a five-factor model showing very strong factor correlations (0.95–1); hence, they concluded that the one-dimensional model revealed the best fit. For the current study, the instrument was translated by a professional translator from English to Norwegian, and then a bilingual associate professor within the research group backtranslated the Norwegian version to English. Small adjustments were made to wording aiming at conceptual equivalence between the English and Norwegian versions of the HLSAC instrument.

### Mental health-promoting knowledge (MHPK-10)

The MHPK-10 was developed in 2017 to measure adolescents’ positive mental HL^25^. The scale consists of ten items assessed on a six-point scale and covers three core theoretical components of good mental health: relatedness, autonomy, and competence^25^. The measure was developed and tested among Norwegian adolescents and found to be valid and reliable for this population^25^. The MHPK-10 was used to test convergent validity in this study, as one can expect that HL and mental HL to some extent are correlated since mental HL has arisen from the domain of HL and must be understood in that context^31^.

### Perceived knowledge to take care of one’s own health

To assess the adolescent’s perceived levels of knowledge needed to take care of their own health, two single items were combined: Item 1: *On a scale from 1 to 5, do you think you have enough knowledge to take care of your physical health? Item 2: On a scale from 1 to 5, do you think you have enough knowledge to take care of your mental health?* These items were self-developed for the study to assess adolescents’ perceived level of knowledge needed to take care of their own health. To assess perceived knowledge of health, the mean score from perceived knowledge of mental and physical heath was calculated for each respondent by summing the scores of both items and dividing by two. This score was used to test convergent validity, as the questions directly asked about adolescents’ perceived knowledge level in regard to taking care of their own health; higher score indicate higher level of perceived knowledge to take care of one’s own health.

### Statistics

Descriptive statistics were performed with IBM SPSS version 27^32^, while confirmatory factor analysis (CFA) was conducted using Stata 17.1^33^. The underlying dimensionality of the data was investigated together with the adequacy of each item. To assess convergent validity, the correlation between HL measured by the HLSAC and positive mental HL measured by the MHPK-10 as well as the correlation between the HLSAC and adolescents’ perceived knowledge to take care of their own health were measured with Spearman’s correlations. For the correlation analyses, the *p* value was set to 1%.

The literature indicates that Cronbach’s α alone cannot be generally trusted as an estimator of reliability (a scale’ internal consistency)^34–37^. Therefore, composite reliability coefficient was additionally estimated utilizing Raykov’s reliability coefficient^38^ which is a measure commonly seen as more accurate than Cronbach’s alpha. Raykov’s reliability coefficient computes coefficients for factors with and without correlated errors^39^, representing a stronger reliability test than the alpha coefficient. A reliability coefficient of ≥ 0.7 is considered good for both coefficients^39–42^. Furthermore, an item analysis was conducted including means, standard deviation, missing along with skewness and kurtosis for each of the items.

CFA is commonly used across clinical research^40,41^, including the development and psychometric evaluation of measurement instruments. CFA is an element of the broader multivariate technique structural equation modeling (SEM) and deals specifically with measurement models^41^. A strength of CFA is that it accounts for random measurement error, yielding a truly accurate evaluation of the psychometric properties of a scale. Hence, using empirical data, CFA aims to confirm a theoretical model (here, the HLSAC)^43^. With the application of CFA, a high loading of an item indicates that the factor and the respective item share common variance^44^. Factor loadings below 0.32 are considered poor, while those ≥ 0.45 are fair, ≥ 0.55 good, ≥ 0.63 very good, and above 0.71 excellent^44^. As a rule of thumb, a minimum loading of 0.32 corresponds to approximately 10% overlapping variance with the other items in the factor^45^.

A range of fit indices are used to assess the relationship between the observed data and the theoretical model, that is, the fit of the measurement model. In line with the rules of thumb given as conventional cutoff criteria^42^, the following fit indices were used to evaluate model fit: chi-square (χ^2^) and its *p* value, which is significant in most cases. Therefore, it is suggested to consider the value of χ^2^/degrees of freedom (df) χ^2^/df (≤ 2 good fit, ≤ 3 acceptable)^46,47^. When inspecting assumptions of normality, both skewness and kurtosis were significant, indicating non-normal distribution of data. Therefore, the Satorra-Bentler-scaled chi-square statistic was applied as a goodness of fit statistics^40,47,48^. Furthermore, the root mean square error of approximation (RMSEA) (≤ 0.05 good fit, ≤ 0.10 acceptable) and the standardized root mean square residual (SRMS) (≤ 0.05 good fit, ≤ 0.10 acceptable), the comparative fit index (CFI) (≥ 0.95 good fit, ≥ 0.90 acceptable) and the Tucker–Lewis index (TLI) (≥ 0.95 good fit, ≥ 0.90 acceptable) were used^42,49^.

## Results

### Descriptive analysis

In total, 1127 of 2811 students (40.1%) at the four schools were given the questionnaire from their class teachers (classwise). Thus, teachers served as gatekeepers for participation at a class level. In total, 1054 of the 1127 students that were given the questionnaire, completed the questionnaire, yielding a response rate of 93.5%. Table 1 lists the sample characteristics; largely, the participants were born in Norway, had parents with higher education and experienced a good family economy. Gender was evenly distributed; approximately half of the respondents were vocational students, and the other half were preparing for higher education. Approximately 60% of the included adolescents’ parents lived together.

**Table 1:** Description of the sample included in the analysis, N = 1028.

	N	Percent (%)
**Gender**		
Female	488	47
Male	527	51
Missing	13	1
**Age**		
16	270	26
17	371	36
18	229	22
19	82	8
20	35	3
21	8	1
Missing	33	3
**Education**		
General studies	510	50
Vocational studies	491	48
Missing	27	2
Parents’ education*		
Primary and lower secondary school	54	5
Upper secondary school	257	25
University, up to 4 years	225	22
University, more than 4 years	228	22
Don’t know	215	21
Missing	49	5
**Family finances**		
Good	737	72
Neither good nor bad	184	18
Bad	67	6
Missing	40	4
**Parents live together**		
Yes	597	58
No	394	38
Missing	37	4
**Born in Norway**		
Yes	932	91
No	79	8
Missing	17	1

*Assessed by asking about each parent; the mean score between the mother and father is presented.

Table 2 presents the item analysis of the HLSAC, including mean, standard deviation, missing, skewness and kurtosis for each item. Further, the excluded items are marked with an x in the last column. As shown, missing is low. Both skewness and kurtosis were significant; all items are negatively skewed showing estimates between -0.42 and -1.01. Negative skew -also referred to as left-skewed- refers to a longer or fatter tail on the left side of the distribution, while positive skew refers to a longer or fatter tail on the right. These two skews refer to the direction or weight of the distribution. Skewness tells us the direction of outliers, but not the amount of them. Furthermore, items 1, 4 and 5 reveal the highest kurtosis, indicating that the variance in these responses is low; most responses are close to the mean score. Finally, all items show a relatively high mean score ranging between 2.98-3.42; the max score is 4.

**Table 2:** Item analysis HLSAC.

Item	N	Mean	SD	Missing %	Skewness		Kurtosis		Excluded
					Std		Std		
					Statistic	Error	Statistic	Error	
1. I have good information about health	1031	3.22	0.68	2.2	− 0.68	0.08	0.73	0.15	
2. When necessary, I am able to give ideas on how to improve health in my immediate surroundings (e.g., a nearby place or area, family, friends)	983	3.02	0.75	6.74	− 0.57	0.08	0.31	0.16	x
3. I can compare health-related information from different sources	981	2.98	0.73	6.92	− 0.42	0.08	0.04	0.16	
4. I can follow the instructions given to me by healthcare personnel (e.g., nurse, doctor)	999	3.42	0.68	5.2	− 1.01	0.08	0.88	0.16	x
5. I can easily give examples of things that promote health	1005	3.42	0.67	4.6	− 1.00	0.08	0.98	0.15	x
6. I can judge how my own actions affect the surrounding natural environment	996	3.29	0.67	5.5	− 0.66	0.08	0.32	0.15	
7. When necessary, I find health-related information that is easy for me to understand	993	3.39	0.68	5.8	− 0.91	0.08	0.67	0.16	
8. I can judge how my behaviour affects my health	997	3.35	0.72	5.4	− 0.89	0.08	0.40	0.16	x
9. I can usually figure out if some health-related information is right or wrong	987	3.04	0.73	6.4	− 0.42	0.08	0.08	0.16	
10. I can give reasons for choices I make regarding my health	987	3.24	0.72	6.4	− 0.73	0.08	0.33	0.16	

Excluded items from the original items HLSAC in the HLSAC-6 are marked with an x in the excluded column.

### Confirmatory factor analysis (CFA)

#### Model 1—the 10-item one-dimensional version

The original ten-item version of the HLSAC scale revealed significant estimates, with factor loadings (λ) between 0.63 and 0.78 and R^2^-values ranging from 0.40 to 0.61. However, the model fit was poor (Table 3). The χ^2^ and RMSEA were too high, while the CFI and TLI were too low, all of which indicated misspecification. Hence, we scrutinized the residuals and the modification indices (MIs). There were no significant residuals but 14 MIs ≥ 10, among which some were extremely high: MI = 90.014 (items 1 and 2), followed by MI = 49.468 (items 4 and 5), MI = 32.933 (items 3 and 8), MI = 30.592 (items 5 and 9), MI = 30.371 (items 9 and 10) and MI = 28.774 (items 4 and 9). Paakkari et al.^11^ developed the HLSAC scale in Finland based on 5 core theoretical dimensions: (1) theoretical knowledge of health issues, (2) practical knowledge, (3) individual critical thinking, (4) self-awareness and (5) citizenship. The original ten items assess each of these five domains. Since the ten-item one-dimensional model showed an extremely poor fit, we tested the 5-factor solution based on Pakkari et al.^11^. Good/acceptable reliability coefficients for the five dimensions (ranging between 0.65 and 0.79), including two items each, as well as a better fit than Model 1, were found. However, this five-factor structure had much too high estimates for the χ^2^ and RMSEA, while the other fit indices were good: χ^2^ = 218.503 (df = 25), χ^2^/df = 8.74, *p* = 0.0001, RMSEA = 0.092, *p* value for test of close fit = 0.0001, CFI = 0.96, TLI = 0.93, and SRMR = 0.034. Consequently, we examined the one-dimensional solution, scrutinizing the items’ reliability and construct validity. Table 3 lists the goodness-of-fit indices along with Raykov’s composite reliability.

**Table 3:** Confirmatory factor analysis of the HLSAC scale. Goodness-of-fit indices.

Fit Measure	Model 1 1-factor N = 920 10 items	Model 2 1-factor N = 933 9 items (2)	Model 3 1 factor N = 937 8 items (2,4)	Model 4 1 factor N = 939 7 items (2,4,5)	Model 5 1 factor N = 944 6 items (2,4,5,8)
χ^2^ *Satorra Bentler*	351.585	236.407	168.697	102.675	34.529
*p* value	0.0001	0.0001	0.0001	0.0001	0.0001
$\frac{{x^{2} }}{{df}}$_*Satorra Bentler*_	10.05 (Df^1^ = 35)	8.76 (Df = 27)	8.43 (Df = 20)	7.33 (Df = 14)	3.84 (Df = 9)
RMSEA	0.099 (CI: 0.090–0.109)	0.091 (CI: 0.081–0.102)	0.089 (CI: 0.077–0.102)	0.082 (CI: 0.068–0.097)	0.055 (CI: 0.036–0.075)
*p value (close fit test)*	0.0001	0.0001	0.0001	0.0001	0.309
SRMR	0.043	0.036	0.033	0.028	0.019
CFI	0.93	0.95	0.96	0.97	0.99
TLI	0.91	0.93	0.94	0.96	0.98
${\rho c}\; = \;\frac{{(\sum {\uplambda })^{2} }}{{\left[ {(\sum {\uplambda })^{2} + \sum ({\uptheta })} \right]}}$	0.91	0.91	0.90	0.89	0.87

*HLSAC *Health Literacy for School-Aged Children measurement model. Removed items in parentheses. *RMSEA* Root mean square error of approximation. *SRMS *Standardized root mean square residual, *CFI* Comparative fit index, *TLI* Tucker–Lewis’s index, ^1^*Df *Degrees of freedom, *ρc* Composite reliability, Raykov’s reliability coefficient.

### Reliability

The reliability of a scale depends on the factor loadings (λ) and the multiple squared correlations (R^2^); the present findings showed standardized factor loadings ranging from 0.63 to 0.78, with R^2^ estimates between 0.40 and 0.61. Both the Cronbach's alpha and composite reliability were 0.91, and the inter-item correlations showed values ranging between 0.40 and 0.65.

### Construct validity of the HLSAC original one-factor model

An inspection of the standardized residuals and the MIs revealed no significant residuals, but several pairs of items showed an extremely high MI. The highest MI estimate was for the pair of items 1 and 2 (MI = 90.014), indicating misspecification. Item 1 concerns “*having good information regarding health*”, while item 2 assesses the “*ability to give examples of things that promote health*”. The interitem correlation was 0.52, indicating that these items overlap and share error variance. Accordingly, to increase the model fit, it is reasonable to let these error terms correlate. However, correlated error terms should be included only with caution^50,51^; this finding may indicate that one of these items is redundant in its current verbalization. Therefore, since item 2 showed a lower loading and R^2^ than item 1, we excluded item 2. Possibly, the wording of this item including “giving ideas on how to improve one’s health in one’s immediate surroundings” may not communicate clearly to this age group. Hence, the wording and relevance of this item for adolescent HL may benefit from being tested with the adolescent age group.

### Further adaptation of the one-factor model: model 2-model 5

**Figure 1:**
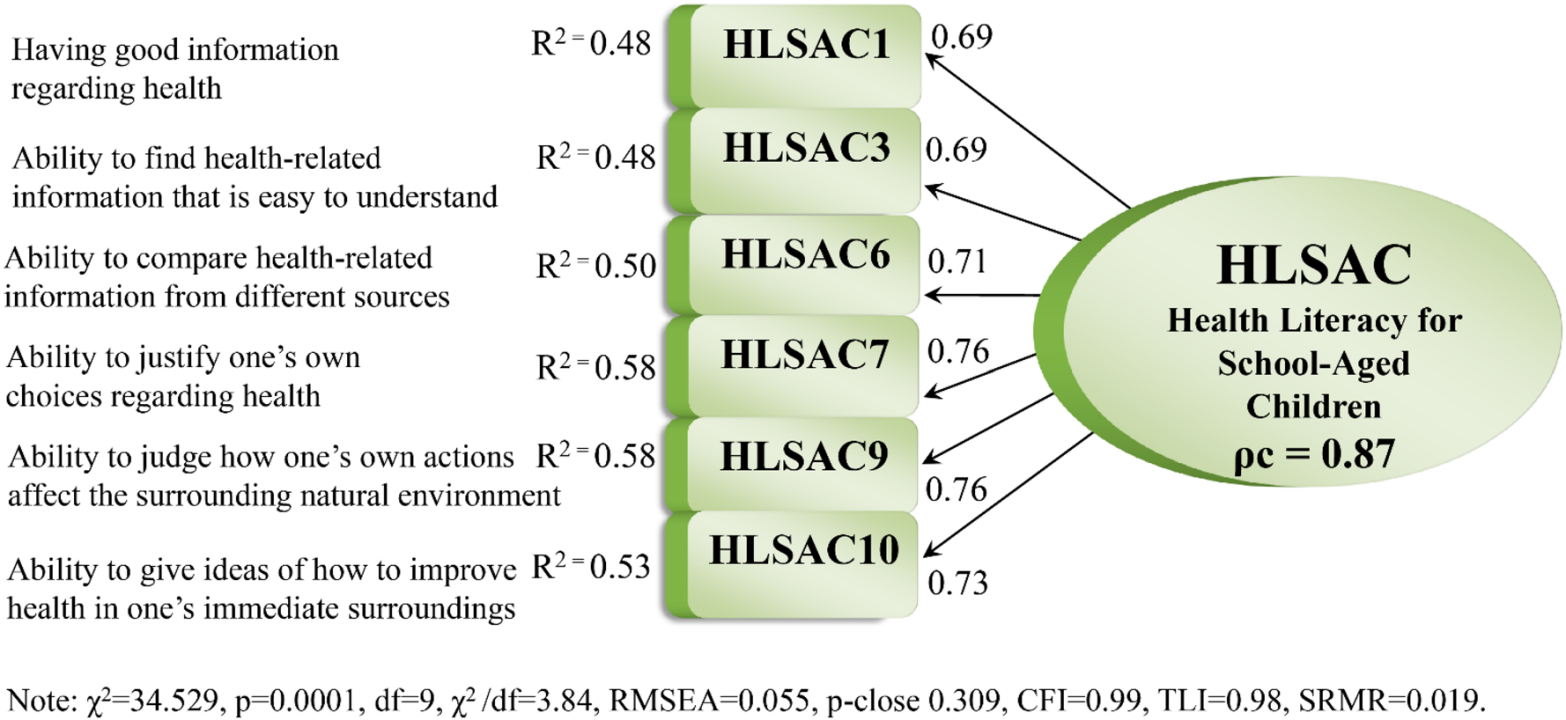
Model 5, the best fitting measurement model of the HLSAC scale, i.e., the HLSAC-6.

With item 2 excluded, Model 2 showed an improved fit, but still the fit was poor (Table 3). However, there were nine MI ≥ 10, among which some presented very high estimates: MI = 32.439, 29.368 and 27.730 for the pair of items 5–9, 5–6 and 9–10, respectively. Considering the MIs, factor loadings, R^2^-values and the theoretical content of the items, we removed item 4 (“*ability to follow the instructions given by doctors and nurses*”) and ran Model 3, including eight of the ten items. For Model 3, the fit was enhanced (Table 3). Nevertheless, the χ^2^ value was still too high, and there was an excessively high estimate for the RMSEA. Accordingly, some items were still troublesome. Model 4 excluded item 5 (“*ability to decide if health-related information is right or wrong”*), which improved the χ^2^ and RMSEA values but not enough. Finally, Model 5, excluding item 8 (“*ability to judge how one’s own behavior affects one’s health*”) and thus including 6 of the 10 original items (items 1, 3, 6, 7, 9, and 10) representing the five original theoretical components of the HLSAC, showed a substantially improved fit (Table 3). Figure 1 portrays the best fitting model (Model 5) including factor loadings, R^2^-values, composite reliability, and model fit indices.

Table 4 presents the means standard deviations, and Spearman’s correlation matrices for the variables HLSAC, HLSAC-6, MHPK-10 and perceived knowledge along with Cronbach’s alpha for HLSAC, HLSAC-6 and MHPK-10. As shown, HLSAC and HLSAC-6 highly correlates with each other, and also correlates significantly and positively with MHPK-10 and Perceived Knowledge, supporting hypothesis 3 (H_3_). Accordingly, convergent validity was supported; this represents an aspect of construct validity and is based on the construct’s relationships to other variables.

**Table 4:** Results of the convergent analysis for HLSAC and HLSAC-6.

*Construct*	*Mean*	*SD*	*Cronbach’s alpha*	*Spearman’s correlation coefficient*		
				*HLSAC*	*MHPK-10*	*Perceived knowledge*^+^
HLSAC	3.25	0.16	0.91	1	0.38*	0.36*
HLSAC-6	3.20	0.18	0.87	0.93*	0.36*	0.32*
MHPK-10	4.28	0.16	0.80	0.38*	1	0.20*
Perceived knowledge^+^	4.23	0.03	–	0.36*	0.20*	1

*Sig at 0.01.

^+^mean score of a two-item questions asking: on a scale from 1 to 5, do you have enough knowledge to take care of your own physical/mental health?

## Discussion

This study aimed to assess the psychometric properties of the Norwegian version of the HLSAC scale among adolescents aged 16–21 years. In doing so, we addressed the dimensionality, reliability, and construct validity of the HLSAC in this population. When evaluating a measurement model, two aspects are important: (1) the underlying dimensionality of the data (not too many, not too few factors) and (2) the adequacy of the individual items. This study assessed how well the original one-dimensional measurement model and the Finnish 5-factor solution of the HLSAC (H_1_) fit the observed data and assessed its reliability and construct validity (H_2_) among Norwegian adolescents.

### Dimensionality (H_1_)

The original 10-item one-dimensional solution showed a poor fit to our data, indicating misspecifications. Thus, since Paakkari et al.^11^ developed the HLSAC with items assessing five core dimensions, we tested whether this five-factor structure would fit better. As shown in the results section, this solution improved the fit, but only moderately: the chi-square and RMSEA values were still very high, pointing at misspecifications. The estimate for χ^2^/df should be ≤ 3 for an acceptable fit and ≤ 2 for a good fit, while Model 1 showed an estimate of χ^2^/df = 10.05. However, regarding the chi-square as a model fit index, there are limitations. First and foremost, chi-square is sensitive to sample size. A misfit may be trivial; however, with larger samples, the p-value decreases, and then there are higher estimates^52^. The present effective sample size is large (N = 920). Considering the sensitivity of the chi-square statistics to sample size, a wide variety of other indices have been suggested to assess model adequacy. This means that in practice, the chi-square test is “not always the final word in assessing fit”^53^. As a minimum, the RMSEA, CFI, and SRMR should be reported in combination with the chi-square^43^. The use of multiple fit indices provides a more holistic view of the goodness of fit, accounting for sample size, model complexity, and other considerations relevant to the study. Concerning the RMSEA, this estimate is found to show lower values with higher numbers of observations, that is, with large sample sizes^54,55^. For Model 1, the RMSEA was too high (0.092), while the CFI, TLI and SRMR suggested a good fit: for an acceptable fit, the RMSEA should be ≤ 0.080^36–38^ or ≤ 0.10^39^, while estimates ≤ 0.050 suggest a good fit. Despite the large sample size, the RMSEA estimate (0.092) was very high. Thus, we interpreted that the poor fit for Model 1 was not associated with the dimensionality but rather related to shared error variance or content validity of the items. As stated in the aim section: if the wording of items is too similar or verbalized so that respondents interpret two items referring to approximately the same aspect, factor loadings, R^2^-values along with reliability coefficients can be very good, however without adding substantially to the content validity. Items interpreted by respondents as simply a rewording of other items are undesirable^30^.

### Reliability

Reliability and construct validity are related to the adequacy of the individual items. The 6-item version of HLSAC includes items which are good indicators of the HL construct among adolescents, with highly significant standardized factor loadings, preferably > 0.71. The square of a standardized factor loading (R^2^), termed the variance extracted of the item, represents how much variation in an item the latent construct explains^37^. Considering the factor loadings and the R^2^ values in Model 1, six (items 1, 5, 6, 7, 8, and 10) of the ten items displayed excellent (≥ 0.71) loadings. The remaining four items (items 2, 3, 4 and 9) showed good loadings ranging between 0.63 and 0.69. Accordingly, the ten items revealed good reliability, explaining much of the variance in the latent construct indicated by good values for Cronbach’s alpha (α) (Table 4) and composite reliability (ρc) (Table 3)^39,42^. The high loadings and correlations between the items along with the strong alpha and composite reliability indicate high internal consistency of the scale.

### Construct validity

Construct validity reveals the accuracy of a measurement, reflecting the extent to which the measurement model tests the hypothesis or theory it is meant to measure^56^. In the present study, convergent validity (H_3_) was supported by a significant positive correlation between both HLSAC, HLSAC-6 and positive mental HL and the adolescents’ perceived level of knowledge needed to take care of their own health (Table 4). However, to achieve a good fit we removed four items. First, item 2 (“*ability to give examples of things that promote health*”) seemed to overlap with item 1 (“*having good information regarding health*”). It is possible that adolescents interpreted that giving examples of things that promote health (item 2) is closely related to having good information regarding health (item 1); basically, they might have found the latter to be covered by item 1. Next, item 4 (“*ability to follow the instructions given by doctors and nurses*”) had very high MIs with items 5 and 9. Probably, these adolescents understood their ability to follow instructions given by health professionals (item 4) as strongly associated with their ability to decide if health-related information is right or wrong (item 5). Presumably, adolescents consider health information given by doctors and nurses to be correct. Therefore, removing item 4 improved the model fit. Also, healthy young individuals aged 16–21 years old attending school might consider following instructions by doctors and nurses to be less applicable to their daily lives. This population is largely healthy and is therefore generally not seeing doctors and nurses as much as the general population or receiving instructions from these professionals. Third, item 5 (“*ability to decide if health-related information is right or wrong*”) was troublesome, involving several very high MIs, indicating that respondents may have perceived the content of this item too be identical to that of other scale items. The same was evident for item 8 (“*ability to judge how one’s own behavior affects one’s health*”). Hence, adolescents aged 16–21 years old feasibly consider judging about right and wrong health-related information, along with judging how one’s own behavior affects one’s health, to characterize unfamiliar ways of thinking about their daily lives and behaviors. Although these items showed good reliability (high loadings/R^2^-values), their theoretical content or wording seemed less pertinent in this population. That is, although reliability was good, the construct validity was limited; items 2, 4, 5 and 8 appeared as possibly redundant or in the need of clarification and precision.

Model 5, including six of the original ten items, presented the best fit to our data: the RMSEA was acceptable and close to good fit (RMSEA = 0.055), and the chi-square was too high (χ^2^/df = 3.84, df = 9); however, as already stated, the χ^2^/df should be ≤ 3 for an acceptable fit and ≤ 2 for a good fit. Nevertheless, considering the limitations of the chi-square related to large samples, accompanied by the other fit indices (RMSEA, CFI, TLI, SRMR) showing very good estimates for Model 5, we concluded that Model 5 is valid and reliable.

In some respects, our results contrast studies in comparable countries such as the recent Danish^21^and Italian^18^ validations of the HLSAC. Including all ten items in one dimension, the Danish study found an excellent fit with factor loadings ranging between 0.52 and 0.75, and good internal consistency (Cronbach's alpha = 0.86), while the Italian study demonstrated an acceptable fit. Though, the Italian χ^2^(df) was 8.72 which is much too high, however accompanied by an acceptable RMSEA (0.08) and a somewhat low CFI (0.92), representing fit indices more in line with the present study. Furthermore, the present fit indices along with the Italian were close to those obtained in other European countries (Poland χ^2^(df) = 168.83(35), *p* = 0.000; RMSEA = 0.08, CFI = 0.93, SRMR = 0.04; and Belgium χ^2^(df) = 69.23(35), *p* = 0.000; RMSEA = 0.07, CFI = 0.92, SRMR = 0.05) in a cross-national study^17^. Like our study, Velasco & Gragnano^18^ tested the five-factor solution showing identical fit indices but demonstrating very strong factor correlations (≥ 0.82). Moreover, similar to our study, the Italian version of item 5 (“ability to follow the instructions given by doctors and nurses”) revealed a low reliability.

The Danish mean age was 12.2 years, including participants attending 6th or 7th grade, while the Italian study involved Lombardian students 13–15 years-old randomly sampled: thus, quite homogeneous samples. The present study included adolescents 16–21 years old representing a less homogeneous and older sample, possibly indicating that the HLSAC is less appropriate in older samples of adolescents. Further, the Danish study presents a comprehensive effort involving face validity of the Danish wording of the items. Hence, possibly the Danish content validity is stronger than the Italian and the Norwegian versions. Authors have argued that the HL construct is content- and context-specific and should be defined differently for such an ever-changing population as adolescents^57,58^. Moreover, there are possibly some minimal cultural differences concerning the HLSAC^17^. Hence, these results may indicate that the HLSAC might be more appropriate for use in the intended age-groups, rather than among older adolescents, and that content validity is important to establish separately in this age-group.

### Strengths and limitations

Significant factor loadings, several goodness-of-fit indices, and a significant correlation in the expected directions with the measures positive mental HL and the adolescents’ perceived level of knowledge needed to take care of their own health supported the psychometric properties of the HLSAC construct. Nevertheless, a good model fit does not guarantee that the “true model” is obtained; other alternative models might fit the data equally well as the identified model^59^. Moreover, the present data were drawn from a relatively homogenous population of suburban Norwegian adolescents; thus, the results may not necessarily be transferable to a more diverse adolescent population in other areas of Norway.

In Model 5, we excluded four out of ten items. Hence, Model 5 eliminates some information, which may be a limitation. Nonetheless, considering adolescents’ motivation to complete a questionnaire, a short, reliable, and valid measure of HL is preferable. The effective (listwise) sample size was N = 920, which is a large sample size, signifying a strength of this study. A rate of 10 cases per observed variable is given as a rule of thumb^39,41^. The models tested in this study included 6–10 items; accordingly, the sample of N ≥ 920 represents a strong power of the statistical tests. In total, 11% of data had missing values, that were deleted listwise. About 40% of the students at the four participating schools were given the questionnaire. One school agreed to include only first year students, and the teachers could choose whether to hand out the questionnaire to their respective classes or not. The response rate among students receiving the questionnaire was very high, and the actual number of responses were relatively high (1054). Furthermore, for classes where teachers chose to not hand out the questionnaire, we have no reason to believe that some particular aspects, except from Norwegian language skills, the distribution of gender, age and study line did not differ from the whole schools’ distribution. A small proportion of the sample (7%) was not born in Norway, however, this is less than the distribution in the general population in Norway (13.6%; Statistics Norway 2022). Hence, our sample is probably not representative of the immigrant population of Norway, and the measurement of HL among adolescent immigrants should therefore be further investigated in future studies.

The HLSAC was translated from English to Norwegian, not from the original Finnish language, being a limitation of this study. Further the wording of the items was not tested with the target population. The current study relies heavily on statistical testing of the items and the model fit indices, implying a data driven item selection. Future studies of the HLSAC for measuring Norwegian adolescents’ HL should include both a thorough translation from Finnish to Norwegian and testing the concept of HL and the items’ wording with the target group; the adolescents themselves, in addition to the statistical testing and model fit indices from the statistical models.

## Conclusions

This study suggests a one-dimensional solution of the HLSAC scale including six of the original 10 items and thus introduces the HLSAC-6 for adolescents aged 16–21. A short, reliable, and valid measure of HL among adolescents is beneficial; thus, the current study introduces the HLSAC-6 consisting of items 1, 3, 6, 7, 9 and 10 from the original HLSAC for assessing HL among Norwegian adolescents in upper secondary schools. The work on assessing HL in the context of adolescents’ health is a continuing process, and the modifications of the instrument indicate the need for further investigation regarding the dimensionality of the instrument. Additional research exploring the psychometric properties of the HLSAC is necessary to establish the generalizability and validity of the reported findings. Moreover, the current study supports further research on measuring HL among adolescents. We suggest that future studies involve adolescents themselves in refining instruments for measuring HL among adolescents, i.e. by conducting cognitive interviews as well as focus-group-discussion on the concept of HL, and testing the wording of the HLSAC items with the adolescents, prior to further psychometric evaluations of both the original 10-item HLSAC and the suggested HLSAC-6. Finally, developing and testing more age-appropriate items for the measurement of HL in this age-group is suggested based on the findings of this study.

## Supplementary Information

Supplementary Information.

## Data Availability

The raw data supporting the findings in this manuscript can be found at the NTNU Norwegian University of Science and Technology, Department of Public Health and Nursing, Trondheim, Norway, and are available from the corresponding author upon reasonable request.

## Abbreviations

CFA: Confirmatory factor analysis
CFI: Comparative fit index
HL: Health literacy
HLSAC: Health Literacy for School-Aged Children
MHL: Mental health literacy
MI: Modification indices
RMSEA: Root mean square error of approximation
SRMR: Standardized root mean square residual
STG: United Nations Sustainable Development Goals
TLI: Tucker–Lewis index
