# Author Correction: Validation of the brief instrument “Health Literacy for School-Aged Children” (HLSAC) among Norwegian adolescents

**DOI:** 10.1038/s41598-023-39368-z

**Published:** 2023-07-27

**Authors:** Hanne Nissen Bjørnsen, Unni Karin Moksnes, Mary‑Elizabeth B. Eilertsen, Geir Arild Espnes, Gørill Haugan

**Affiliations:** 1 Department of Public Health and Nursing, Norwegian University of Science and Technology, Postbox 8905, 7491 Trondheim, Norway; 2 NTNU Center for Health Promotion Research, Norwegian University of Science and Technology, 7491 Trondheim, Norway; 3 Faculty of Nursing and Health Science, Nord University, 7601 Levanger, Norway

Correction to: *Scientific Reports* https://doi.org/10.1038/s41598-022-26388-4, published online 21 December 2022

The original version of this Article contained errors in Table 2, where the values given for “N”, “Mean”, “SD”, “Skewness” and “Kurtosis” were incorrect. The correct and incorrect values appear below.

Incorrect:

**Table:** 

Item	N	Mean	SD	Missing %	Skewness		Kurtosis		Excluded
					Std		Std		
					Statistic	Error	Statistic	Error	
1. I have good information about health	1043	4.53	0.81	1.0	− 1.83	0.08	4.09	0.15	
2. When necessary, I am able to give ideas on how to improve health in my immediate surroundings (e.g., a nearby place or area, family, friends)	1039	1.6	1.22	1.4	2.19	0.08	3.96	0.15	x
3. I can compare health-related information from different sources	1042	4.39	.088	1.1	− 1.41	0.07	2.28	0.15	
4. I can follow the instructions given to me by healthcare personnel (e.g., nurse, doctor)	1041	4.64	0.95	1.2	− 2.63	0.08	1.57	0.15	x
5. I can easily give examples of things that promote health	1037	4.38	0.91	1.6	− 1.04	0.08	1.57	0.15	x
6. I can judge how my own actions affect the surrounding natural environment	1021	4.42	0.86	2.4	− 1.02	0.08	1.48	0.15	
7. When necessary, I find health-related information that is easy for me to understand	1042	4.42	0.86	2.4	− 1.02	0.08	1.48	0.15	
8. I can judge how my behaviour affects my health	1035	4.69	0.77	1.8	− 2.24	0.08	6.17	0.15	x
9. I can usually figure out if some health-related information is right or wrong	1035	4.37	0.98	1.8	− 1.45	0.08	2.02	0.15	
10. I can give reasons for choices I make regarding my health	1022	4.52	0.80	3.0	− 1.29	0.07	2.58	0.15	

Correct:

**Table:** 

Item	N	Mean	SD	Missing %	Skewness		Kurtosis		Excluded
					Std		Std		
					Statistic	Error	Statistic	Error	
1. I have good information about health	1031	3.22	0.68	2.2	− 0.68	0.08	0.73	0.15	
2. When necessary, I am able to give ideas on how to improve health in my immediate surroundings (e.g., a nearby place or area, family, friends)	983	3.02	0.75	6.74	− 0.57	0.08	0.31	0.16	x
3. I can compare health-related information from different sources	981	2.98	0.73	6.92	− 0.42	0.08	0.04	0.16	
4. I can follow the instructions given to me by healthcare personnel (e.g., nurse, doctor)	999	3.42	0.68	5.2	− 1.01	0.08	0.88	0.16	x
5. I can easily give examples of things that promote health	1005	3.42	0.67	4.6	− 1.00	0.08	0.98	0.15	x
6. I can judge how my own actions affect the surrounding natural environment	996	3.29	0.67	5.5	− 0.66	0.08	0.32	0.15	
7. When necessary, I find health-related information that is easy for me to understand	993	3.39	0.68	5.8	− 0.91	0.08	0.67	0.16	
8. I can judge how my behaviour affects my health	997	3.35	0.72	5.4	− 0.89	0.08	0.40	0.16	x
9. I can usually figure out if some health-related information is right or wrong	987	3.04	0.73	6.4	− 0.42	0.08	0.08	0.16	
10. I can give reasons for the choices I make regarding my health	987	3.24	0.72	6.4	− 0.73	0.08	0.33	0.16	

As a result, in the Results section, under the subheading ‘Descriptive analysis’,

“As shown, missing is low except from item 10 disclosing more missing than the other items. Both skewness and kurtosis were significant; most items are negatively skewed showing estimates ranging between − 1.02 and − 2.24, whereas only item 2 is positively skewed. Negative skew -also referred to as lef-skewed- refers to a longer or fatter tail on the left side of the distribution, while positive skew refers to a longer or fatter tail on the right. These two skews refer to the direction or weight of the distribution. Skewness tells us the direction of outliers, but not the amount of them. Furthermore, items 1, 2 and 8 reveal the highest kurtosis, indicating that the variance in these responses are low; most responses are close to the mean score. Finally, all items show a relatively high mean score ranging between 4.37 and 4.69; only item 2 had a low mean of 1.6.”

now reads:

“As shown, missing is low. Both skewness and kurtosis were significant; all items are negatively skewed showing estimates between − 0.42 and − 1.01. Negative skew -also referred to as left-skewed- refers to a longer or fatter tail on the left side of the distribution, while positive skew refers to a longer or fatter tail on the right. These two skews refer to the direction or weight of the distribution. Skewness tells us the direction of outliers, but not the amount of them. Furthermore, items 1, 4 and 5 reveal the highest kurtosis, indicating that the variance in these responses is low; most responses are close to the mean score. Finally, all items show a relatively high mean score ranging between 2.98 and 3.42; the max score is 4.”

Additionally, under the subheading ‘Construct validity of the HLSAC original one-factor model’,

“Therefore, since item 2 showed a lower loading and R2 than item 1, we excluded item 2. Moreover, this item disclosed a significant positive skewness (Table 2). In a positively skewed distribution, most values on the graph are on the left side, and the curve is longer towards the right trail. In this study, this indicates that several adolescents recorded this item close to the mean, while some scored it considerably higher; considering the extensively lower mean for item 2 this is rational. How can this low mean for item 2 be explained? Possibly, the wording of this item including “giving ideas on how to improve one’s health in one’s immediate surroundings” may not communicate clearly to this age group. Hence, the wording and relevance of this item for adolescent HL may benefit from being tested with the adolescent age group.”

now reads:

“Therefore, since item 2 showed a lower loading and R2 than item 1, we excluded item 2. Possibly, the wording of this item including “giving ideas on how to improve one’s health in one’s immediate surroundings” may not communicate clearly to this age group. Hence, the wording and relevance of this item for adolescent HL may benefit from being tested with the adolescent age group.”

Finally, in the Discussion section, under the subheading ‘Construct validity’,

“In the present study, convergent validity (H3) was supported by a significant positive correlation between both HLSAC, HLSAC-6 and positive mental HL and the adolescents’ perceived level of knowledge needed to take care of their own health (Table 2).”

now reads:

“In the present study, convergent validity (H3) was supported by a significant positive correlation between both HLSAC, HLSAC-6 and positive mental HL and the adolescents’ perceived level of knowledge needed to take care of their own health (Table 4).”

The original Article has been corrected.

