# Supplementary material for: Validation of the brief instrument “Health Literacy for School-Aged Children” (HLSAC) among Norwegian adolescents
Source: Sci Rep. 2022 Dec 21;12:22057. doi: 10.1038/s41598-022-26388-4 (PMC9772172; doi:10.1038/s41598-022-26388-4)
Supplement: Supplementary file 1 — Supplementary Information. [file 41598_2022_26388_MOESM1_ESM.pdf]

Husk: Bare ett kryss på hvert spørsmål.

3. Jeg er sikker på at jeg …

	Helt feil 1	*Litt feil* 2	*Litt riktig* 3	*Helt riktig* 4
1. … har god kunnskap om helse	☐	☐	☐	☐
2. … kan vurdere helserelatert informasjon fra ulike kilder	☐	☐	☐	☐
3. … kan vurdere hvordan mine handlinger påvirker miljøet	☐	☐	☐	☐
4. … kan finne forståelig informasjon om helse når jeg trenger det	☐	☐	☐	☐
5.. … vanligvis kan avgjøre om helserelatert informasjon er rett eller feil	☐	☐	☐	☐
6. … kan begrunne valg jeg tar når det gjelder helsen min	☐	☐	☐	☐

From the following options, choose the one that best describes your opinion

**I am confident that…**	Not at all true	Not completely true	Somewhat true	Absolutely true
1. I have good information about health	❑	❑	❑	❑
2. I can compare health-related information from different sources	❑	❑	❑	❑
3. I can judge how my own actions affect the surrounding natural environment	❑	❑	❑	❑
4. When necessary I find health-related information that is easy for me to understand	❑	❑	❑	❑
5. I can usually figure out if some health-related information is right or wrong	❑	❑	❑	❑
6. I can give reasons for choices I make regarding my health	❑	❑	❑	❑
